# At-Home Training With a Rhythmic Video Game for Improving Orofacial, Manual, and Gait Abilities in Parkinson’s Disease: A Pilot Study

**DOI:** 10.3389/fnins.2022.874032

**Published:** 2022-06-13

**Authors:** Frédéric Puyjarinet, Valentin Bégel, Christian Geny, Valérie Driss, Marie-Charlotte Cuartero, Valérie Cochen De Cock, Serge Pinto, Simone Dalla Bella

**Affiliations:** ^1^University of Montpellier, Montpellier, France; ^2^Department of Psychology, McGill University, Montreal, QC, Canada; ^3^Department of Geriatrics, CHRU of Montpellier, Montpellier, France; ^4^Clinical Investigation Centre, CHRU of Montpellier, Montpellier, France; ^5^Aix Marseille Univ., CNRS, LPL, Aix-en-Provence, France; ^6^Department of Neurology, Beau Soleil Clinic, Montpellier, France; ^7^International Laboratory for Brain, Music and Sound Research (BRAMS), Montreal, QC, Canada; ^8^Department of Psychology, University of Montreal, Montreal, QC, Canada; ^9^Centre for Research on Brain, Language and Music (CRBLM), Montreal, QC, Canada; ^10^University of Economics and Human Sciences in Warsaw, Warsaw, Poland

**Keywords:** Parkinson’s disease, rhythmic training, music-based interventions, mobile technologies, serious video games

## Abstract

Rhythm disorders are consistently reported in Parkinson’s disease (PD). They manifest across motor domains, such as in orofacial (oral diadochokinesis), manual (finger tapping), and gait tasks. It is still unclear, however, whether these disorders are domain- and task-specific, or result from impaired common mechanisms supporting rhythm processing (general dysrhythmia). We tested the possibility that an at-home intervention delivered via a rhythmic video game on tablet improves motor performance across motor domains in PD. Patients with PD (*n* = 12) played at home a rhythmic video game (Rhythm Workers) on tablet, in which they finger-tapped to the beat of music, for 6 weeks. A control group (*n* = 11) played an active non-rhythmic video game (Tetris). A third group (*n* = 10) did not receive any intervention. We measured rhythmic abilities in orofacial, manual and gait motor domains, as well as rhythm perception, before and after the intervention. Patients who performed the rhythmic training improved their orofacial and manual rhythmic performance. This beneficial effect was linked to improved rhythm perception only following the rhythmic training period. We did not observe any improvement in rhythmic abilities in the other two groups. In this pilot study, we demonstrated that at-home intervention with a rhythmic video game using finger tapping can have beneficial effects on motor performance across different motor domains (manual and orofacial). This finding provides evidence of a general dysrhythmia in PD and paves the way to technology-driven interventions aiming at alleviating rhythm-related motor deficits in PD.

## Introduction

Beyond cardinal motor symptoms of Parkinson’s disease (PD), severe rhythm deficits encompassing different motor domains are consistently reported, potentially representing an early marker of the disease (Cochen De Cock et al., 2020). These deficits manifest in heightened rhythmic variability of upper-limb, orofacial, and gait coordination (Skodda et al., 2010; Dalla Bella et al., 2017; Lowit et al., 2018). Rhythm deficits are not confined to motor tasks. They also extend to rhythm perception, such as detecting a beat in a rhythmic auditory sequence in the absence of motor output (Grahn and Brett, 2009; Benoit et al., 2014). These disorders of rhythm production across motor effectors and poor beat perception are highly correlated in PD (Puyjarinet et al., 2019). A parsimonious explanation of rhythm deficits in PD is referred to as *general dysrhythmia* or *unique dysrhythmia* (Cantiniaux et al., 2010; Skodda et al., 2010; Tolleson et al., 2015). According to this hypothesis rhythm perception and production disorders in PD would stem from the impairment of common brain mechanisms underpinning rhythm perception and production. These mechanisms include distributed neuronal networks involving both sub-cortical areas (the basal ganglia and the cerebellum) and cortical regions (e.g., the SMA) (Repp and Su, 2013; Cannon and Patel, 2021). Impairment of such basal-ganglia-cortical networks is consistent with the pathophysiology of PD (Factor and Weiner, 2008; Wichmann, 2019).

Though appealing, the hypothesis of *general dysrhythmia* in PD was tested only in a handful of correlational studies. For example, it is known that gait and speech rhythmic features (walking cadence and speech rhythmicity) are tightly linked (Cantiniaux et al., 2010), and that motor rhythmic variability in PD is closely related across different effectors—upper and lower limbs (finger tapping, gait), and the orofacial system (oral diadochokinesis) (Puyjarinet et al., 2019). Most importantly, we demonstrated that the severity of rhythm deficits across such motor domains can be predicted by the degradation of rhythm perception (Puyjarinet et al., 2019). This intriguing finding confirmed the impairment of a central mechanism devoted to rhythm processing, independent of motor control *per se*. Nevertheless, it is still unknown whether a general rhythm impairment plays a causal role in motor and perceptual rhythm deficits in PD.

We examined this possibility in the present pilot study by testing whether the beneficial effects of a rhythm-based intervention on a given effector (i.e., manual) do transfer to other effectors (orofacial, gait). Interventions targeting rhythmic skills can take different forms, such as rhythmic auditory cueing (Ghai et al., 2018; Dalla Bella, 2020), full-body physical activities (e.g., dance, Hackney and Earhart, 2009; Hsu et al., 2022), or serious games (Bégel et al., 2017, 2018). A serious game is a video game combining serious intentions, such as educational, professional or medical purposes, with pleasant and recreational aspects (Rego et al., 2010, 2014; Marsh, 2011). The advantage of serious video games is being designed for targeting specific functions such as attentional/executive, motor, or rhythmic abilities (Wiemeyer and Kliem, 2012; Bégel et al., 2018). In this study, we trained auditory-motor rhythmic skills *via* finger tapping in a group of PD patients with a dedicated serious video game we developed (*Rhythm Workers*, Bégel et al., 2018). This serious video game was validated in PD in a previous study (Dauvergne C. et al., 2018). As controls, we trained a second group of patients with a non-rhythmic classic video game (*Tetris*, Green and Bavelier, 2003; Nouchi et al., 2012), while a third group received no intervention. To test the effect of the type of training, we compared patients’ motor performance in the orofacial, manual, and gait domains, and their rhythm perception before and after intervention.

## Materials and Methods

### Patients

Thirty-three PD patients (6 women; mean age: 68.40 ± 7.32 years; age range: 50–82), participated in the experiment. PD was diagnosed from 2 to 25 years prior to the experiment (mean duration: 10.13 ± 5.69 years) in accordance with the UK Brain Bank criteria (Gibb and Lees, 1988). Patients were under optimal medication (60–90 min after their usual morning dose), which was stable for at least 4 weeks prior to the start of the experiment. This study was approved by the appropriate Committee (*Comité de Protection des Personnes*, *Centre Hospitalier Universtaire*, Montpellier: CPP N°2015-A01090-49). All participants signed an informed consent in accordance with the Helsinki Declaration (World Medical Association, 2013). Motor, perceptual, demographic, clinical characteristics, and cognitive status are provided in Table 1.

**TABLE 1:** Patients’ demographic and clinical characteristics.

	Rhythm workers		Tetris		No intervention group		*P*-value
	Mean (range)	*n*	Mean (range)	*n*	Mean (range)	*n*	
**Demographics**							
Age (years)	66.8 (50–77)	12	67.7 (67–56)	11	70.3 (63–79)	10	0.46
Men/women	–	8/4	–	10/1	–	9/1	0.50
Disease duration (years)	9.9 (2–25)	12	9.6 (2–23)	11	10.2 (6–18)	10	0.21
**Clinical characteristics**							
**MDS-UPDRS**							
Total score	65.4 (32–102)		57.0 (24–88)		56.7 (34–79)		0.50
Motor subscore (part 3)	29.0 (3–51)		26.2 (10–39)		29.9 (20–46)		0.74
Speech item (3.1)	1.4 (0–3)		(0–2)		1.1 (0–3)		0.51
Upper limb items (3.4a–3.6b)	7.1 (0–16)		4.7 (0–10)		8.1 (1–14)		0.14
Lower limb items (3.7a–3.8b)	6.5 (0–13)		5.1 (2–9)		6.3 (0–12)		0.54
Hoehn and Yahr score	2.2 (2–3)		2.1 (2–3)		2.2 (2–3)		0.88
**Cognitive assessment**							
MoCA	25.7 (22–30)		25.0 (19–28)		25.7 (20–30)		0.77

*P-values are results of one-way ANOVAs comparing the three groups on each measure. MDS-UPDRS, Movement Disorder Society—Unified Parkinson’s disease Rating Scale (Goetz et al., 2008); MoCA, Montreal Cognitive Assessment (Nasreddine et al., 2005).*

### Experimental Design

In the absence of previous studies using rhythmic training in patients with PD using serious video games, we calculated the minimal sample size for our pilot study based on a previous study run in our laboratory using standard auditory cueing (Benoit et al., 2014; PD patients, *n* = 15; controls, *n* = 10). In that study patients with PD were submitted to a 1-month rhythmic training with auditory cueing, leading to beneficial effects on rhythm perception and performance, as well as gait. PD patients were randomly assigned to one of the three experimental arms: (*i*) *Rhythm Workers* (rhythm training group), (*ii*) *Tetris* (non-rhythm control training group), and (*iii*) no intervention group. Assignment to the groups was achieved using the free online application Group Maker (available athttps://livecloud.online/en/group-maker) that enables generating sequences of random numbers. The patients were instructed to play the games (*Rhythm Workers* or *Tetris*) using a tablet device (LG^©^ G Pad 8.0 model) for at least 30 min, 4 times a week, during a period of 6 weeks ± 2 days. For checking whether participants followed these instructions, the number and duration of training sessions, as well as the performance with each of the games were automatically logged on the tablet at each session to be further compared between training types. Patients who played with *Rhythm Workers* were asked to finger tap to the beat of auditory rhythmic stimuli of variable complexity (metronome and musical excerpts) (Bégel et al., 2018; Dauvergne C. et al., 2018). The alignment between finger taps and the beat of rhythmic stimuli served to provide a feedback on the performance, and to progress through difficulty levels: the greater the synchronization of the taps to the beat, the higher the score, then allowing to move on to the next difficulty level. Patients in the *Tetris* group were required to play this classic game as an active control condition (Green and Bavelier, 2003; Nouchi et al., 2012). No specific, active, involvement was asked to the patients of the third group. A [post-pre]intervention design was used to assess orofacial, gait, manual, and perceptual rhythmic performance in the three groups.

### Assessment of Rhythmic Skills

We tested rhythmic abilities with widely used orofacial, manual, gait and perceptual rhythmic tasks. To test orofacial rhythmic ability the patients performed an oral diadochokinesis task (i.e., oral sequential motion rate, namely the repetition of the tri-syllable pseudoword *pataka* for 30 s, at fast rate). We recorded the patients’ productions with a digital recorder (Zoom H4SP^©^). Audio files were pre-processed and analyzed using Praat software (Boersma and van Heuven, 2001). We assessed manual and perceptual rhythmic skills using the *Battery for the Assessment of Auditory Sensorimotor and Timing Abilities* (BAASTA, Dalla Bella et al., 2017). The patients performed two manual rhythmic tasks from BAASTA: unpaced and paced finger tapping tests. In unpaced tapping, the patients’ task was to finger tap regularly at a comfortable rate for 60 s in the absence of a pacing stimulus, while maintaining the tapping rate as constant as possible. In paced tapping, the patients had to synchronize their taps to the beat of a musical excerpt. In the rhythm perception task (Beat Alignment Test—BAT, Iversen and Patel, 2008; Dalla Bella et al., 2017), the patients had to judge whether a sequence of tones was aligned or not with the beat of short musical excerpts. BAASTA tests were administered using a tablet device (LG^©^ G Pad 8.0 model), and auditory stimuli were delivered over headphones (Sennheiser^©^ HD201). Finally, we tested gait rhythmicity by asking patients to walk along a computerized walkway (GAITRite^©^ 301 system), at their preferred rate, for a distance of 8 m. To avoid variability (accelerations and decelerations) at the onset of the gait trial, participants started walking 2 m before the starting edge of the walkway and continued walking 2 m after the end of the walkway. Patients performed this task three times, and data were averaged. We have studied and described more extensively these tasks elsewhere (Dauvergne C. et al., 2018; Puyjarinet et al., 2019; Cuartero M. C. et al., 2021).

### Variables and Analyses

We measured patients’ rhythmic performance by computing the variability, namely the standard deviation (SD) of inter-event intervals in the rhythmic tasks: inter-vowel intervals (IVIs) for *orofacial rhythmic variability*; inter-tap intervals (ITIs) for *manual rhythmic variability*; and stride time intervals (STIs) for *gait rhythmic variability* (see Puyjarinet et al., 2019 for details). IVIs, ITIs, and STIs variabilities account for the quality of motor coordination in each rhythmic domain, with higher scores indicating worse performances (greater variability or lower regularity).

For assessing rhythm perception, we calculated a beat perception score obtained in the rhythm perception task (*d*-prime, Dalla Bella et al., 2017). The higher the score, the better the performance. For both intervention groups (i.e., *Rhythm Workers* and *Tetris* groups), data were converted into *Z*-scores relatively to the performance of the no intervention group.

To test the effect of interventions on rhythmic variability across the different motor domains, we used mixed-design ANOVAs to compare the performance of the *Rhythm Workers* and *Tetris* groups, where Session (i.e., pre- vs. post-intervention) was the within-subject factor, and Group (*Tetris* vs. *Rhythm Workers*) the between-subject factor. We applied the Holm correction for multiple comparisons. We further assessed whether demographic, clinical, and cognitive variables would influence rhythmic performance by successively including them as covariates in the ANOVAs. In addition, to compare the effects of interventions on motor rhythmic performances with those on rhythm perception, we calculated [post-pre]intervention difference scores. We compared the two groups with a one-tailed Welch’s *t*-test. Moreover, one-tailed Pearson correlations between [post-pre]intervention difference scores for motor and perceptual tests were calculated. Most data were normally distributed according to the Shapiro–Wilk test, and whenever it was not the case, non-parametric tests were used. Statistics were computed using *JASP* software (JASP Team, 2016). All significant effects were set at *P* ≤ 0.05.

## Results

**FIGURE 1:**
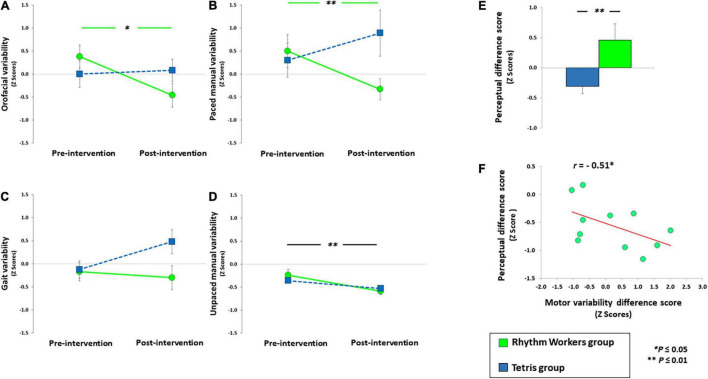
Performance of patients playing *Rhythm Workers* and *Tetris* in the rhythmic oral diadochokinesis **(A)**, paced manual **(B)**, gait **(C)**, and unpaced manual **(D)** tasks. **(E,F)** The post- minus pre-intervention difference score in the rhythm perception task, for each group. A positive difference score indicates an improvement of rhythm perception following the intervention. **(F)** The relation between an improvement in rhythm perception (perceptual difference score) and the change in rhythmic variability post- minus pre-intervention averaged across motor domains (post-motor variability difference score) for the *Rhythm Workers* group. A negative motor variability difference score indicates an improvement.

Descriptive statistics for rhythmic performance assessed pre- and post-intervention are summarized in Table 2 and the effects of interventions on rhythmic performance are presented in Figure 1. Note that the three groups were comparable in terms of motor and perceptual rhythmic performance at baseline (all *P*s > 0.19 when comparing the three groups’ performance with ANOVAs). On average the patients that trained with *Tetris* spent more time in playing the game (mean = 21.3 h, range = 1.39–44.32 h) than patients trained with *Rhythm Workers* (mean = 4.8 h; range = 1.07–15.07 h) (*W* = 18, *P* < 0.01; rank-biserial correlation = –0.72).

**TABLE 2:** Rhythm performance for PD patients in the two experimental groups (*Rhythm Workers* and *Tetris*) and in the no intervention group.

	Rhythm workers group			Tetris group			No intervention group		
Rhythmic domain	Pre-int. Mean (*SD*)	Post-int. Mean (*SD*)	*n*	Pre-int. Mean (*SD*)	Post-int. Mean (*SD*)	*n*	Pre-int. Mean (*SD*)	Post-int. Mean (*SD*)	*n*
**Orofacial**									
IVIs (ms)	193.0 (30.5)	189.6 (40.5)	12	173.4 (33.8)	159.1 (35.7)	11	205.4 (36.2)	183.1 (17.9)	10
IVI SD	55.6 (18.7)	45.0 (19.9)	12	47.2 (21.0)	57.2 (17.7)	11	47.2 (21.3)	55.3 (22.0)	10
**Manual**									
Paced ITI (ms)	420.8 (124.3)	498.9 (134.5)	12	478.9 (106.5)	466.5 (117.8)	11	517.7 (127.3)	575.9 (60.8)	10
Paced ITI SD	101.2 (70.0)	37.3 (37.4)	11	88.8 (71.5)	98.8 (72.2)	10	63.2 (58.1)	49.5 (45.0)	10
Unpaced ITI (ms)	580.2 (197.9)	487.7 (96.4)	12	550.8 (179.6)	548.7 (99.8)	9	868.0 (520.7)	666.5 (284.7)	10
Unpaced ITI SD	130.4 (164.2)	48.9 (70.5)	12	88.3 (58.4)	66.2 (68.1)	9	214.4 (345.5)	221.2 (288.3)	10
**Gait**									
STI (ms)	1165.5 (218.5)	1163.1 (180.6)	12	1150.0 (81.5)	1124.4 (202.8)	11	1135.4 (138.8)	1139.8 (135.4)	9
STI SD	29.0 (7.6)	27.1 (6.0)	10	31.8 (7.1)	26.1 (10.3)	11	30.1 (17.6)	30.6 (15.8)	9
**Rhythm perception**									
d’	2.2 (1.4)	2.5 (0.9)	11	1.3 (0.5)	1.0 (0.5)	11	1.9 (0.6)	2.0 (0.7)	10

*ITIs, inter-taps interval; IVI, inter-vowels interval; STI, inter-strides interval.*

*Due to outliers (i.e., individuals with performance of ± 3 SD), or missing data for some patients who could not complete all the tasks because of health issues or fatigue, n differ across tasks.*

### Rhythmic Motor Performance

Both ANOVAs on orofacial and paced manual rhythmic variability showed a Session x Group interaction [*F*_(1,21)_ = 6.04, *P* = 0.02, η^2^ = 0.19; Figure 1A; *F*_(1,21)_ = 5.74, *P* = 0.02, η^2^ = 0.23; Figure 1B]. Patients in the *Rhythm Workers* group showed lower orofacial rhythmic variability [*t*_(11)_ = 2.55, *P* = 0.01, *d* = 0.73] and lower manual tapping variability [*t*_(11)_ = 2.50, *P* = 0.01, *d* = 0.75] after the intervention than before. No effect of intervention was found in the *Tetris* group on orofacial (*P* = 0.70) and manual rhythmic variability (*P* = 0.86). Both *Tetris* and *Rhythm Workers* reduced the unpaced manual rhythmic variability [Session effect: *F*_(1,19)_ = 13.67, *P* < 0.01, η^2^ = 0.39; Figure 1D]. In the same task, there was neither a Session × Group interaction, nor a main effect of Group (*P*s > 0.21). When considering the effect of rhythmic training on gait rhythmic variability, the main effects of Session and Group were not significant (*P*s > 0.12), and the Session x Group interaction failed to reach significance [*F*_(1,19)_ = 3.55, *P* = 0.07, η^2^ = 0.14; Figure 1C]. Patients in the group without intervention did not show any change in their rhythmic variabilities, whatever the motor domain and the time of testing (all *P*s > 0.32 in orofacial, manual, and gait domains).

### Rhythm Perception, Correlation With Motor Performance, and Clinical Tests

The two interventions differed in their effect on rhythm perception [*t*_(15.63)_ = 2.62, *P* < 0.01, *d* = 1.09] (Figure 1E). Playing with *Rhythm Workers* improved patients’ rhythm perception (*Z* = 1.62, *P* = 0.05, *d* = 0.46), unlike playing with *Tetris* (*W* = –1.08, *P* = 0.14). The reduction of rhythmic variability in the motor domains, whatever the effector, was linked to an improvement in rhythm perception in the *Rhythm Workers* group only (*r* = –0.51; *P* = 0.05; Figure 1F), whereas it was not the case in the *Tetris* group (*r* = 0.36; *P* = 0.84). Considering the age, disease duration, MDS-UPDRS total and motor scores, Hoehn and Yahr stage, and MoCA score as covariates in the aforementioned ANOVAs did not modify the results relative to orofacial, manual (paced and unpaced) and gait rhythmic variability (Session x Group interactions remained significant in orofacial and manual domains, with *P*s < 0.05, and all *P*s = n.s. in the gait domain).

### Clinical Findings

For the MDS-UPDRS motor subscore (part 3), the interaction between the effect and type of intervention failed to reach significance [Session × Group interaction: *F*_(1,21)_ = 3.51, *P* = 0.07, η^2^ = 0.12]. Due to the marginally significant interaction, we ran further analyses showing overall less severe motor symptoms, as showed by lower MDS-UPDRS motor subscores (part III), in patients who played *Rhythm Workers* [*t*_(11)_ = 2.57, *P* = 0.02, *d* = 0.74] relative to their performance pre-intervention. We found no such difference in the *Tetris* patient group (*P* = 0.96). We did not find similar trends for MDS-UPDRS specific items related to speech (3.1), upper (3.4a–3.6b) and lower (3.7a–3.8b) limbs, for none of the patient groups.

## Discussion

In this pilot study, we provide first evidence that an at-home intervention with a rhythmic video game improves rhythm performance (i.e., it reduces motor variability) in PD patients, when evaluating both manual and orofacial motor tasks. We do not observe any improvement with a comparable non-rhythmic video game (i.e., *Tetris* group), and in absence of training (i.e., no intervention group). These effects persist after controlling for patients’ clinical characteristics such as severity of motor symptoms, disease duration, and cognitive functioning. Critically, we demonstrate that the motor improvement is linked to enhanced rhythm perception resulting from the rhythmic training. The effect of rhythmic training is even more surprising considering that patients played the rhythmic video game less time than the non-rhythmic one. Overall, we provide first causal evidence in support of a general dysrhythmia in PD, in keeping with our previous work (Puyjarinet et al., 2019). Showing that the beneficial effects of rhythmic training can transfer from one effector to the others paves the way to innovative clinical applications.

Which cerebral mechanisms are likely to underpin the observed transfer effect of rhythmic training across motor domains? A pivotal notion in explaining this finding is the concept of predictive timing, namely the ability to predict accurately the time of occurrence of an upcoming event (e.g., the next syllable, or the next step), based on the regular temporal structure of a sequence (Large and Jones, 1999; Piras and Coull, 2011; Schwartze and Kotz, 2013). The mechanisms underlying predictive timing engage distributed subcortico-cortical pathways, including the basal ganglia, the cerebellum, the supplementary motor area, and parietal and dorsal-premotor cortices (Coull and Nobre, 2008; Kotz and Schwartze, 2011; Piras and Coull, 2011; Schwartze and Kotz, 2013). Rhythmic interventions are likely to engage these networks, such as in gait rehabilitation *via* rhythmic auditory cueing (Dalla Bella, 2020), which is also found to improve PD patients’ rhythm performance both in motor and perceptual tasks (Benoit et al., 2014; Ghai et al., 2018). This form of auditory rhythm-based intervention is likely to capitalize on relatively spared predictive timing (Dalla Bella et al., 2017; Dalla Bella, 2018), which would improve as a result of the training (as shown in a rhythm perception task), and drive beneficial effects across different motor effectors (Puyjarinet et al., 2019; Dalla Bella, 2020). This hypothesis is in keeping with the results of a previous study (Puyjarinet et al., 2019) showing that motor variability in PD is correlated across motor domains and can be predicted by the performance in a beat perception task, thus irrespective of the specific motor output. The causal evidence that rhythm perception can be improved selectively by rhythmic training, as opposed to training in a non-rhythmic game, and that this improvement is related to reduced motor variability across domains indicates that the beneficial effects of rhythmic training may be driven by a central predictive mechanism relatively independent of the motor output.

Our results do not show improvement in rhythmic performance tested in a gait task after the intervention. This finding may be surprising considering that rhythm-based interventions are supposed to improve gait parameters in PD, notably speed and stride length (for reviews, Ghai et al., 2018; Janzen et al., 2019; Dalla Bella, 2020). Unlike gait cadence (Janzen et al., 2019), rhythm variability assessed in the current study appeared to be a variable less sensitive to such intervention in gait. Nevertheless, rhythm variability was the most appropriate measure to consider for cross-domain (oral, manual, and gait) comparison. We could compute the same measures of timing for motor events and their variability across domains, and we already showed that these measures are correlated in PD (Puyjarinet et al., 2019). Another possibility to explain the lack of improvement on gait rhythmic variability following the rhythmic training may be the explicit nature of the rhythmic game. The game targets sensorimotor synchronization, namely an explicit timing task with the goal of producing finger taps aligned to the beat of musical sequences. Explicit timing tasks rely on basal-ganglia-cortical networks, and can be considered as a form of “cognitive controlled timing.” Conversely, spontaneous gait tested in the present study may rely more on “implicit timing” mechanisms, where temporal information is not targeted by the task *per se*, and can be used to optimize performance in non-temporal tasks (Coull and Nobre, 2008; Avanzino et al., 2016). Implicit timing critically relies on cerebello-thalamo-cortical pathways, with parietal cortex involvement (Lewis and Miall, 2003; Avanzino et al., 2016). This dissociation between explicit and implicit timing neural mechanisms could at least partially account for difference between the effect of our intervention on rhythm variability in gait, oral and manual tasks. Our findings suggest that training in an explicit timing task using finger tapping may not transfer to spontaneous gait rhythmic variability. Future studies should compare the effects of training timing abilities using an implicit task vs. an explicit task on gait.

Other explanations may rely on the nature of the video game training that does not require the participants to walk. Finger tapping may have not been sufficient to observe cross-effector beneficial effects for gait, while walking in real condition to the rhythm of metronome or music beat is known to induce cross-effector effects including spontaneous gait, at least for spatio-temporal gait features such as speed and stride length (Janzen et al., 2019). In the future, it would be interesting to increase the duration of the rhythmic training or its intensity, as more than 6 weeks of practice may be needed to induce beneficial effects in gait (i.e., reduce rhythm variability).

We also found a tendency of rhythmic training to improve the global motor state of patients, as assessed by the MDS-UPDRS. If confirmed, this tendency could have important clinical consequences. A relevant example of clinical application would be to use rhythmic video games in the context of a motor rehabilitation program. Serious games are an evolving field demonstrating already promising results for healthcare (Rego et al., 2014), and in motor rehabilitation in PD (Perrochon et al., 2019). Using games like Rhythm Workers holds a lot of promise for devising at-home, auto-rehabilitation programs widely accessible and low-cost. Many advantages can be associated with the practice of such serious games, among which the motivating and funny nature of the training. Feedback from patients who played with Rhythm Workers mainly concerned the challenging and playful aspects of the game. Another advantage of training at home is that there are no constraints associated to traveling to rehabilitation centers (e.g., travel time, fatigue). Several times a week, the patient can practice independently, in a safe environment, under the supervision of a therapist who can provide a personalized program at distance. Integrating rhythmic training sessions *via* serious game could complement the effects of more traditional rehabilitations while avoiding muscular fatigue due to repetitive physical effort (e.g., gait rehabilitation programs for preventing the risk of falling) (Hausdorff et al., 2001). The regular practice of a rhythmic video game at home may alleviate symptoms such as altered orofacial coordination—which typically manifests in PD hypokinetic dysarthria (Atkinson-Clement et al., 2015; Lowit et al., 2018).

Yet, there are still challenges that will motivate further research. Notably, adherence to the program is central to the success of an intervention (Perrochon et al., 2019), and even more important in serious video games than in standard clinical interventions, owing to the paucity of guidelines. Thus, adherence of the specific serious video game protocol should be evaluated on a case-by-case basis. In the present experiment, and despite PD patients benefited from the rhythmic training, about 60% of them were far from reaching the required playing time. The fact that some players did not reach the minimum time required could be explained by reasons independent of the characteristics of the game. Several patients showed cognitive deficits associated with PD, which may have induced fatigue and may explain their reduced adherence to the intervention. These issues will have to be considered in future studies to increase patients’ adherence to the rhythmic training game. For example, an individualized approach adapting the game features (e.g., its level of difficulty) to the patient’s spared cognitive capacities and rhythmic abilities (Dalla Bella et al., 2018; Dotov et al., 2019; Dalla Bella, 2020) is likely to increase adherence to the intervention. Complementary to this approach is the assessment of rhythmic abilities prior to the intervention, so as to identify the patients who are the most likely to benefit from the intervention, and those who may experience adverse effects (Cochen De Cock et al., 2018). Finally, one of the advantages related to the current health context (i.e., the COVID-19 epidemic) is to allow patients to take part in the rehabilitation program remotely without contact with other people, thus limiting the risk of infection. In sum, there are several points in favor of using rhythmic serious video games in home-based therapeutic practice, in addition to more traditional approaches. One of the limitations of our study is the small sample size, which is typical of pilot studies. In spite of this limitation, the results were very promising, and the observed effect sizes were moderate to large. The pilot study will pave the way to a randomized clinical trial in which a larger sample of patients will be considered. Second, in our study we measured the improvement of the rhythmic variability due to the intervention in behavioral tests, while lacking an investigation of the underlying brain changes and involved neuronal mechanisms. Our planned future investigation will include an assessment of structural and functional brain changes before and after the intervention, in order to highlight the contribution of the perceptual and motor regulation loops involved in rhythmic performance. Longitudinal studies with follow-up assessments on larger cohorts of patients will help also to investigate potential long-term effects of rhythmic training *via* serious video games and their effect on social activities (e.g., conversational skills, daily commuting).

To conclude, we demonstrated in this pilot study that an at-home intervention especially designed for training rhythmic skills with a serious video game improves rhythmic performance in PD, in both manual and orofacial domains, as well as rhythm perception. Therefore, serious games played at home may present a highly promising and cost-effective solution for complementing more traditional motor rehabilitation protocols for PD patients. Finally, from a more clinical point of view, our study provides additional arguments for considering dysrhythmia as an important feature to be more systematically assessed in PD.

## Data Availability Statement

The raw data supporting the conclusions of this article will be made available by the authors, without undue reservation.

## Ethics Statement

The studies involving human participants were reviewed and approved by the Comité de Protection des Personnes Sud-Méditerranée I Marseille, France (CPP N°2015-A01090-49). The patients/participants provided their written informed consent to participate in this study.

## Author Contributions

FP, VB, CG, VD, M-CC, VCD, SP, and SD substantially contributed to the design of the experiment, revised the work, approved the completed version, and addressed the questions related to the accuracy and integrity of the work. FP, VB, CG, and SD conducted the experiments. FP and SD analyzed the results. FP, SP, and SD contributed to writing the manuscript. All authors contributed to the article and approved the submitted version.

## Conflict of Interest

The authors declare that the research was conducted in the absence of any commercial or financial relationships that could be construed as a potential conflict of interest.

## Publisher’s Note

All claims expressed in this article are solely those of the authors and do not necessarily represent those of their affiliated organizations, or those of the publisher, the editors and the reviewers. Any product that may be evaluated in this article, or claim that may be made by its manufacturer, is not guaranteed or endorsed by the publisher.
